# A Rare Manifestation of Tuberculosis Presenting in the United States

**DOI:** 10.1155/2016/8216040

**Published:** 2016-12-22

**Authors:** Osman Bhatty, David Waters, Nicholas Wilka, Shradha Samuel, John Horne, Renuga Vivekanandan

**Affiliations:** Department of Medicine, Creighton University, 601 N 30th Street, Omaha, NE 68131, USA

## Abstract

A 64-year-old Bangladeshi female presented to her primary care physician with a tender right breast lump that had been present for 4-5 days along with subjective fevers and malaise. Initial biopsy revealed granulomas, but Ziehl-Neelsen and Gram stain were negative for TB so antibiotics were prescribed for abscess until culture came positive for tuberculosis. She was started on triple therapy for extrapulmonary tuberculosis, an exceedingly rare presentation that requires high clinical suspicion in the Western world.

## 1. Introduction

Tuberculosis is an age-old disease that can present in any organ in the human body. The two major clinical classifications of tuberculosis associated with this case study are pulmonary tuberculosis (PTB) and extrapulmonary tuberculosis (EPTB). PTB includes infections of the lung parenchyma that can be spread through droplets. In the United States, there have been approximately two hundred and fifty thousand cases of tuberculosis infections (both PTB and EPTB) from 1993 to 2006. Of these, 19% have been associated with extrapulmonary sites. This 19% of EPTB includes lymphatic, pleural, bone/joint, genitourinary, meningeal, peritoneal, and unclassified areas. Breast tuberculosis is included in the unclassified subset and makes up approximately 0.1% of EPTB in the developed world. In areas of higher incidence, breast tuberculosis has been reported to be as high as 4% of EPTB infections [1]. We present a case of breast tuberculosis and management.

## 2. Case Presentation

A 64-year-old female presented to her primary care physician with a tender right breast lump that had been present for 4-5 days. Additionally, she had been feeling feverish and fatigued. Further history indicated that she recently moved to the United States from Bangladesh. Her primary care physician proceeded to perform an ultrasound needle guided biopsy. Biopsy showed abscess formation and necrotizing granulomatous inflammation. Ziehl-Neelsen and Gram stains were negative for any organisms. Patient diagnosed with an abscess and prescribed a course of oral antibiotics. Several weeks later, cultures from the site grew tuberculosis. The patient was travelling abroad at this time and was unable to follow-up. Upon returning to the United States, the patient was informed of the diagnosis of tuberculosis but was unable to obtain an appointment with an infectious disease specialist in her city of residence. A couple months later the patient presented to our infectious disease clinic complaining of daily fevers and a nonhealing lesion on her right breast. She was not complaining of any other symptoms at that time.

Physical exam revealed that she was afebrile but tachycardic. Breast exam was significant for a 1-inch movable mass on the right anterior axillary line without overlying skin changes, warmth, erythema, or nipple changes. Physical exam was otherwise unremarkable. The patient was prescribed oral antituberculosis therapy and was counseled on the need for her family members to be tested for tuberculosis as well. However, the patient's condition worsened over the next couple days and required admission to the hospital. During her hospital admission, her creatinine was found to be elevated from her baseline and she was started on IV fluids. A chest X-ray was obtained and did not reveal any cavitary lesions. A CT scan of the chest revealed nonspecific enlarged axillary, mediastinal, and breast lymph nodes; evidence of prior granulomatous disease of the lungs; no evidence of consolidation, pleural effusion, or pneumothorax; and a diffusely enlarged thyroid with multiple nodules. We were unable to obtain sputum cultures during this admission as the patient was vomiting.

The patient was started on oral pyrazinamide, isoniazid, and rifampin in the hospital. Over the course of three days, the patient showed marked improvement in her symptoms and was discharged on antituberculosis therapy.

## 3. Discussion

Breast tuberculosis is a disease that occurs much more frequently in women of reproductive age and who are multiparous. However, it should be suspected in any patient with a breast mass or abscess with or without a draining sinus irrespective of sex. It may also involve ipsilateral axillary lymph nodes. Breast tuberculosis is hard to diagnose and the index of suspicion must be high. PCR and radiological techniques are crucial to differentiate between tuberculosis versus malignancy [1].

Public health protocols have been diligently assessing the rates of PTB due to the potential to infect persons exponentially through droplets. Such infrastructure has initiated the placement of significant protocols, all of which have a direct effect in reducing rates of PTB and an indirect effort in reducing EPTB. The numbers of active tuberculosis infections (both PTB and EPTB) have decreased in number since 1993. PTB has decreased more in comparison to EPTB and, therefore, the proportion of EPTB has increased to approximately 19% of all recorded tuberculosis infections from 1993 to 2006 [2].

In the 1950s, Mckeown and Wilkinson [3] first classified tuberculosis of the breast into five pathologic types. These types were based on clinical presentations and their microscopic descriptions. While all types have histologically similar features, such as caseating granulomas and necrosis, they differ in their presentation and progression of breast involvement. In many cases of breast tuberculosis or tuberculous mastitis, it is not known how the mycobacterium entered the breast tissue. The leading theory in most cases is that it spread to the breast via retrograde lymphatic flow from ipsilateral lymph nodes [4]. This theory has been supported by studies showing 50–75% of cases having positive lymph nodes ipsilaterally [4]. However, other causes of spread include hematogenous, direct inoculation through the breast ductal system, and contiguous spread from the chest wall, pleura, and so forth.

The first and most common type is Nodular Tuberculous Mastitis [1, 3]. This manifestation of TB presents often as a slow growing solitary mass. It is often a single nodule in one involved breast but may be bilateral. Histologically, this type is characterized by extensive caseating necrosis within the nodule itself and very little fibrosis [1, 3]. This nodule will continue to grow and cause necrosis until it progresses to involve the skin causing sinus tracts to form. Importantly, this type may present early on and bring up concerns of fibroadenoma or carcinoma.

The next type, and second most common, is the Disseminated Tuberculous Mastitis [1, 3]. This type involves the entire breast as multiple tubercles that all intercommunicate. These tubercles are often full of caseating necrosis. Once again, this necrosis often leads to ulceration of the skin and formation of sinus tracts. Epidemiologic studies have shown that this type is more common in older patients [5]. Clinically, this type may be mistaken for aggressive carcinoma due to its complete involvement and ulceration.

The third type is Sclerosing Tuberculous Mastitis [1, 3]. This represents an exaggerated inflammatory response to the bacteria in the breast. Instead of caseating necrosis, the inflammatory reaction causes extensive fibrosis and destruction of breast tissue. Histologically, this type can be very difficult to diagnose due to the lack of caseation and extensive fibrosis. In some cases, fibrosis has been so extensive it has involuted the breast. Once again this type is more common in older patients and is clinically similar to inflammatory or aggressive carcinoma [1].

The fourth and fifth types are much more uncommon. Tuberculous Mastitis Obliterans presents as a cystic mastitis with multiple cystic spaces found throughout the breast [3]. It is thought that this type somehow seeds the ductal system of the breast causing proliferation of ductal epithelium. This proliferation causes obstruction of the ducts leading to cystic formation. Histologically, there is also much periductal fibrosis. The last type that can be associated with any organ system is Secondary Tuberculous Mastitis or Miliary Tuberculosis [3]. Like in all types of miliary spread, breast tissue may be involved with multiple seeds of disease. Unlike the disseminated type earlier, however, they need not be intercommunicating and it is always secondary from a separate primary site.

In more recent times, different classification systems have been proposed to better subtype breast tuberculosis to what is more commonly seen to aid in diagnosis. Tewari and Shukla [6]. Suggest that breast tuberculosis be lumped into three categories instead of specific pathologic types because it is more clinically useful. They suggest lumping all cases into three types: Nodular variants, Disseminated variants, and abscess variants [6]. The first two categories mainly represent the same groups as the original 5-category system mentioned above. The last group of abscess variants is a newer proposed group based on the increased prevalence of cases where an abscess was the original presentation. Epidemiologic studies have shown this to be an increased cause of breast tuberculosis in younger women, particularly those who are breastfeeding or have in recent years delivered a child.

Using this newer system of classification, our patient would be categorized as having a nodular variant. On exam, her presentation suggested a palpable nonfluctuating mass that after biopsy had extensive caseating granulomas with necrosis and surrounding inflammatory response without fibrosis. Although it did not progress naturally, after its removal, a sinus tract formed and persisted until her presentation to our team despite multiple rounds of antibiotics and wound dressings.

It is also worth mentioning the difficulty of securing an infectious disease follow-up in the patient's home town of rural Nebraska. Due to long wait times, she came to the academic center for continued care which was tedious and likely increased caregiver burden. The Association of American Colleges has predicted a shortage of physicians moving forward and the field of infectious disease may be particularly susceptible. This is in light of lower match percentages of internal medicine graduates into the fellowship, which has now long been noted as an issue. Patients in rural areas will continue to have difficulty finding providers unless more graduates choose to subspecialize in the field of infectious disease. The discharge of our patient involved the public health nurse of the local county communicating with their hometown public health nurse to set up further care. This included tuberculosis testing of the immediate family and further evaluation. The state was also notified of this case.

## 4. Diagnosis

The most reliable and definitive method of diagnosing tuberculosis is through bacterial culture of the tissue or Ziehl-Neelsen stain. Unfortunately, the yield of organisms is low in these cases, with bacilli only being isolated in about 25% of cases and AFB being identified in about 12% [5]. In light of this, some authors have endorsed a diagnosis of tuberculosis mastitis with demonstration of caseating granulomas and lymph node involvement [5, 7, 8]. This holds true in endemic countries and empiric treatment with anti-TB drugs should be considered [7, 8]. Fine needle aspiration can be used to obtain samples for histological diagnosis and can diagnose 72% of cases when both epithelial cell granulomas and necrosis are present [9, 10]. Overall, pathological examinations are more valuable than bacteriological examinations and are preferred for the accurate diagnosis of breast TB [11]. Waiting for cultures to return positive can delay diagnosis much like in our own patient so patient background is vital to determine who should receive empiric treatment and how closely they should be followed.

A number of authors have suggested the use of excision biopsy over FNA to rule out differentials [5, 12, 13] and also because adequate tissue samples may not be achieved with only FNA. The differential diagnosis of breast TB includes idiopathic granulomatous mastitis (GM), sarcoidosis, abscess, Wegener's granulomatosis, and giant cell arteritis, as well as other infections like actinomycosis and fat necrosis [14]. An important differentiation is to rule out idiopathic granulomatous mastitis since the treatment of steroids for this would cause harm to those who actually have breast TB. Our patient was treated for abscess with antibiotics until cultures turned positive after several weeks. High clinical suspicion is necessary in high risk individuals.

In light of the difficulty of detecting AFB (more than 10,000 organisms/mL required), nucleic acid amplification test could be very helpful in establishing the diagnosis of TB in smear-negative samples [1]. Two direct amplification tests (DATs) have been approved by the FDA so far. These are the* M. tuberculosis* direct test (MTD) and the Amplicor* M. tuberculosis* test. These can detect directly from clinical samples offering better accuracy than AFB and greater speed than cultures [15]. The appropriateness of its use however remains to be determined as specificity and sensitivity reach 100% and 96%, respectively, in AFB positive smears but vary significantly in AFB smear-negative samples depending on the pretest probability [16]. The primary advantage of these tests is that a positive result to establish a diagnosis can be returned within 24 hours. The United States Centers for Disease Control and Prevention (CDC) has published recommendations for the use of these tests in the diagnosis of TB [17].

Finally, PCR testing is currently not approved but validated internally by laboratories by written protocol. In one study, performing PCR on tissue samples after FNA helped identify 13/26 cases originally reported as granulomatous inflammation on cytology [18], showing PCR may have a role in diagnosis in the future.

Anti-TB treatment is the mainstay in the management of breast tuberculosis but is controversial. Most current guidelines recommend the same regimen for both pulmonary TB and extrapulmonary tuberculosis and studies on the latter are not as robust considering its relative rarity [19]. The treatment is for 6 months and results in a good clinical response [15]; it consists of 2 months of the intensive phase (isoniazid, rifampicin, pyrazinamide, and ethambutol) followed by 4 months on two drugs (isoniazid and rifampicin) [4, 20]. Most series have reported a success rate of medical therapy to be approximately 95% [20]. In some case series, up to 14% of patients required surgical excision due to draining cold abscess, lack of response, or ulcerations [5, 21].

Extrapulmonary tuberculosis is a rare disease, even more so in United States. Gold standard for diagnosis remains culture, but a high index of suspicion is necessary in countries in which TB is not endemic. Fine needle aspiration remains an excellent tool for demonstrating histology compatible with tuberculosis. When this is achieved, anti-TB therapy is the mainstay of therapy with studies showing high success rates.

## Learning Points

1. Tuberculosis of the breast is a rare presentation especially in the West and empiric treatment should be considered in those patient from endemic countries.
2. The treatment is for 6 months and results in a good clinical response; it consists of 2 months of the intensive phase (isoniazid, rifampicin, pyrazinamide, and ethambutol) followed by 4 months on two drugs (isoniazid and rifampicin).
3. Fine needle aspiration remains an excellent tool in confirming histology compatible with diagnosis.

## References

[1] Baharoon S. (2008). Tuberculosis of the breast. *Annals of Thoracic Medicine*.

[2] Peto H. M., Pratt R. H., Harrington T. A., LoBue P. A., Armstrong L. R. (2009). Epidemiology of extrapulmonary tuberculosis in the United States, 1993–2006. *Clinical Infectious Diseases*.

[3] Mckeown K. C., Wilkinson K. W. (1952). Tuberculous disease of the breast. *British Journal of Surgery*.

[4] Shinde S. R., Chandawarkar R. Y., Deshmukh S. P. (1995). Tuberculosis of the breast masquerading as carcinoma: a study of 100 patients. *World Journal of Surgery*.

[5] Tewari M., Shukla H. S. (2005). Breast tuberculosis: diagnosis, clinical features & management. *Indian Journal of Medical Research*.

[6] Tewari M., Shukla H. S. (2005). Breast tuberculosis: diagnosis, clinical features and management. *Indian Journal of Medical Research*.

[7] Kakkar S., Kapila K., Singh M. K., Verma K. (2000). Tuberculosis of the breast: A Cytomorphologic Study. *Acta Cytologica*.

[8] Mehrotra R. (2004). Fine needle aspiration diagnosis of tuberculous mastitis. *Indian Journal of Pathology and Microbiology*.

[9] Kakkar S., Kapila K., Singh M. K., Verma K. (2000). Tuberculosis of the breast: a cytomorphologic study. *Acta Cytologica*.

[10] Martínez-Parra D., Nevado-Santos M., Meléndez-Guerrero B., García-Solano J., Hierro-Guilmain C. C., Pérez-Guillermo M. (1997). Utility of fine-needle aspiration in the diagnosis of granulomatous lesions of the breast. *Diagnostic Cytopathology*.

[11] Sen M., Gorpelioglu C., Bozer M. (2009). Isolated primary breast tuberculosis—report of three cases and review of the Literature. *Clinics*.

[12] Fujii T., Kimura M., Yanagita Y., Koida T., Kuwano H. (2003). Tuberculosis of axillary lymph nodes with primary breast cancer. *Breast Cancer*.

[13] Gupta D., Rajwanshi A., Gupta S. K., Nijhawan R., Saran R. K., Singh R. (1999). Fine needle aspiration cytology in the diagnosis of tuberculous mastitis. *Acta Cytologica*.

[14] Sen M., Gorpelioglu C., Bozer M. (2009). Isolated primary breast tuberculosis-report of three cases and review of the Literature. *Clinics*.

[15] Venyo A. K.-G., Venyo L. K.-G., Maloney D. J. L., Khan A. N. (2015). Tuberculosis of the kidney and the genitourinary tract—a review of the literature. *Hamdan Medical Journal*.

[16] Wobeser W. L., Krajden M., Conly J. (1996). Evaluation of roche amplicor PCR assay for *Mycobacterium tuberculosis*. *Journal of Clinical Microbiology*.

[17] Centers for Disease Control and Prevention (CDC) (2009). Updated guidelines for the use of nucleic acid amplification tests in the diagnosis of tuberculosis. *Morbidity and Mortality Weekly Report (MMWR)*.

[18] Nalini G., Kusum S., Barwad A., Gurpreet S., Arvind R. (2015). Role of polymerase chain reaction in breast tuberculosis. *Breast Disease*.

[19] Lee J. Y. (2015). Diagnosis and treatment of extrapulmonary tuberculosis. *Tuberculosis and Respiratory Diseases*.

[20] Jalali U., Rasul S., Khan A., Baig N., Khan A., Akhter R. (2005). Tuberculous mastitis. *Journal of the College of Physicians and Surgeons Pakistan*.

[21] Elsiddig K. E., Khalil E. A. G., Elhag I. A. (2003). Granulomatous mammary disease: ten years' experience with fine needle aspiration cytology. *International Journal of Tuberculosis and Lung Disease*.

